# High-concentration carbamide peroxide can reduce the sensitivity caused by in-office tooth bleaching: a single-blinded randomized controlled trial

**DOI:** 10.1590/1678-7757-2017-0573

**Published:** 2018-05-07

**Authors:** Aline Carvalho PEIXOTO, Savil Costa VAEZ, Natalia Andrade de Resende PEREIRA, Carla Nogueira da Silva SANTANA, Karla Danielly Alves SOARES, Ana Clara Teles Roriz ROMÃO, Lorena Fernandes FERREIRA, Paulo Ricardo Saquete MARTINS-FILHO, André Luis FARIA-E-SILVA

**Affiliations:** 1 Universidade Federal de Sergipe, Programa de Pós-Graduação em Ciências da Saúde, Aracaju, Sergipe, Brasil.; 2 Universidade Federal de Sergipe, Curso de Odontologia, Aracaju, Sergipe, Brasil.

**Keywords:** Dental esthetics, Tooth bleaching, Tooth bleaching agents

## Abstract

**Objectives:**

A single-blinded, randomized, parallel clinical trial evaluated the use of 37% carbamide peroxide (CP) on bleaching effectiveness and tooth sensitivity reported by patients undergoing in-office tooth bleaching, in comparison with the results of using 35% hydrogen peroxide.

**Material and Methods:**

Forty patients were allocated to receive two sessions of in-office tooth bleaching using either 35% hydrogen peroxide (HP) or 37% CP. Each patient’s sensitivity level was evaluated during and up to 24 h after bleaching. The effectiveness of the bleaching procedures was evaluated with a spectrophotometer one week after each session and 30 days after the last session. The impact of tooth bleaching on the patients’ perceptions regarding smile changes, in addition to the bleaching procedures and their results, were also recorded. Absolute and relative sensitivity risks were calculated. Data on sensitivity level were analyzed using the Mann-Whitney or T-test, and data from the color evaluation were subjected to 2-way repeated measures ANOVA.

**Results:**

The use of CP reduced the risk and level of tooth sensitivity to values close to zero, whereas the difference between the bleaching agents disappeared after 24 h. An increased bleaching effect was observed for HP, mainly due to an improved reduction of redness and yellowness. Participants perceived improved tooth bleaching for HP and reduced sensitivity for CP, but no differences regarding the comfort of the techniques were noted.

**Conclusions:**

In our study, 37% CP resulted in reduced tooth sensitivity but decreased the tooth bleaching effectiveness. However, both bleaching agents resulted in high levels of patient satisfaction.

## Introduction

Tooth bleaching is a non-invasive technique to solve aesthetic complaints from patients regarding their smile. The technique consists in the application of peroxide-based bleaching agents over the buccal surface of discolored teeth. Radicals produced by the peroxide breakdown oxide the organic components of the dental tissue, resulting in whiter teeth^9^ ^,^ ^15^. The bleaching agent can be applied by clinicians using an in-office technique (commonly hydrogen peroxide at high concentrations) or delivered by trays filled with less concentrated peroxide (typically carbamide peroxide) using an at-home bleaching technique. Despite the high success rate of both techniques in bleaching discolored teeth, the tooth sensitivity reported by patients is the most common adverse effect related to the bleaching procedure, especially when high-concentrated hydrogen peroxide is used^8^ ^,^ ^19^ ^,^ ^27^.

Tooth sensitivity is the result of peroxide and its products reaching the pulp chamber, resulting in an inflammatory response of the pulp tissue^1^ ^,^ ^17^ ^,^ ^22^. Clinical trials have demonstrated that the average absolute risk of tooth sensitivity is approximately 51% and 63% for at-home and in-office bleaching techniques, respectively^28^. The lower incidence of tooth sensitivity using at-home techniques is associated with the reduced concentration of peroxides in the bleaching agents used by this technique^8^ ^,^ ^23^ ^,^ ^28^. In contrast with at-home techniques, during an in-office bleaching higher concentrations of peroxides are used to compensate for the reduced time of tooth exposure to the bleaching agent. However, previous studies demonstrated that the use of lower concentrations (20%) of hydrogen peroxide for in-office techniques yields bleaching effects like those obtained with more concentrated peroxide (approximately 35%)^22^ ^,^ ^28^. However, hydrogen peroxide used at low concentrations (e.g., 6%) yields reduced color changes despite the lower incidence of tooth sensitivity^4^.

Several bleaching agents with different hydrogen peroxide concentrations are now available on the market for in-office techniques, including carbamide peroxide-based agents^16^ ^,^ ^20^. Carbamide peroxide is commonly used for at-home bleaching using a tray-based technique and dissociates into hydrogen peroxide (approximately a third of its former concentration) and urea, which further breaks down into water and ammonia^7^ ^,^ ^29^. This last reaction increases the pH of the solution, reducing the enamel demineralization, and the proteolytic activity of urea can improve the bleaching effectiveness. Regarding tooth sensitivity, this adverse effect is strongly related to the presence of peroxides and their sub-products reaching the pulpal chamber to activate TRAP1 (transient receptor potential cation channel with ankyrin domain-type 1)^17^. This activation occurs by peroxide oxidization of cysteines residues in TRAP1 and reaction of oxidizing agent with Fe_2+_ via Fenton reaction^17^. Therefore, reduced tooth sensitivity can be expected using carbamide peroxide since lower concentration of hydrogen peroxide is available. Among the products available on the market, carbamide peroxide-based whiteners with concentrations as high as 35% have been indicated for at-home bleaching procedures^7^. Theoretically, a 35% carbamide peroxide bleaching agent has the same bleaching effect as another agent with approximately 12% hydrogen peroxide that could be used in-office with reduced chair-time^29^.

Moreover, despite the importance of measuring color and tooth sensitivity, differences in these outcomes occasionally do not affect the level of patient satisfaction. In fact, most clinical trials fail to assess the patient’s perception regarding the procedures and their results^11^. Therefore, the aim of this study was to evaluate the effectiveness of a 37% carbamide peroxide bleaching agent used for in-office techniques compared with the results of using 35% hydrogen peroxide. The tested hypothesis was that 37% carbamide peroxide results in reduced color change and tooth sensitivity. Patients’ perceptions regarding the bleaching procedures and their results were also assessed.

## Material and methods

This clinical trial was approved (CAAE: 50511415.1.0000.5546) by the scientific review committee and the committee for the protection of human subjects of the local university. Study protocol was registered at clinicaltrials.gov under No. NCT02935114.

### Study design

This study was a randomized, single-blinded clinical trial, with a parallel design, comparing two tooth bleaching agents. Patients included were submitted to two in-office bleaching sessions using 35% hydrogen peroxide or 37% carbamide peroxide with a delay of one week between the sessions. The study was conducted at the school of Dentistry of the local university from March to September 2016.

### Inclusion and exclusion criteria

The patients included in the study were at least 18 years old, had good general and oral health, and had upper canines darker than the 2.5 M2 tab on the VITA Bleachedguide 3D-MASTER^®^ (Vita-Zahnfabrik, Bad Säckingen, Germany) scale. Participants were recruited by advertisements attached to boards on university buildings. Patients with caries, restorations, severe discoloration (e.g., stains caused by tetracycline), enamel hypoplasia, gingival recession, dentin exposure, visible cracks on buccal enamel, pulpitis, or endodontic treatment on any of the six upper anterior teeth were excluded. Participants who had undergone a previous bleaching procedure, presented prior tooth sensitivity, continuously used anti-inflammatory or analgesic drugs, were smokers, had parafunctional habits, used oral removable or fixed orthodontic appliances, or were pregnant or breastfeeding, were excluded.

### Sample size calculation

The sample size calculation was based on an absolute risk of 90% tooth sensitivity as a primary outcome, as reported in a previous study using a similar bleaching agent (35% HP) and protocol (three 15-min applications)^24^. The calculation was performed for a superiority trial with a binary outcome considering a power test of 80%, a significance level of 5%, and a decrease of 40% for the experimental treatment (37% CP) compared with the control in a parallel design as clinically relevant. Thus, 40 patients (20 *per* experimental condition) were included in the study according to the sample size calculation.

### Random sequence generation and allocation concealment

A randomized list was computer-generated by a person not involved in the intervention or evaluation. The sequence of allocation was inserted into sealed envelopes numbered from 1 to 40 that were opened by the operator only at the time of the intervention. The patients were numbered according to the sequence of enrollment.

### Baseline measurements

To standardize the area of color measurement during the entire experiment, an index was generated by obtaining an upper arch impression with polyvinyl siloxane. A 6-mm-diameter perforation was generated on the index corresponding to the buccal faces of canines to allow the placement of the spectrophotometer (Easyshade Compact Advance 4.0, Vita-Zahnfabrik, Bad Säckingen, Germany) tip. The values of L* (brightness), a* (hue in the red-green axis), and b* (hue in the blue-yellow axis) on canines were measured in triplicate, and the average of the measurements was recorded.

### Intervention

Dental prophylaxis was performed with pumice and water using a rubber cup, and the patients underwent the following procedures according to the treatment allocation:

Hydrogen peroxide - A light-polymerized resin dam (Top Dam, FGM, Joinville, SC, Brazil) was applied over the gingival tissue corresponding to the teeth to be bleached. A 35% hydrogen peroxide-based bleaching agent (Whiteness HP Maxx, FGM, Joinville, SC, Brazil) was mixed, applied over the buccal surfaces of the teeth, and kept in position for 15 minutes. After this time, the bleaching agent was replaced until three applications were completed in the same session.

Carbamide peroxide - No gingival dam was used, and the 37% carbamide peroxide (Power Bleaching, BM4, Palhoça, SC, Brazil) was applied to the buccal surfaces of teeth for 40 minutes. Cotton rolls were used to avoid lip contact with the bleaching agent.

The bleaching agents were used according to the manufacturers’ instructions. After the time determined for peroxide exposure for each protocol, the bleaching agents were removed, and the enamel was polished with felt disks. A second session of bleaching was performed after one week, following the same procedures described previously.

### Evaluations

The tooth sensitivity reported by patients was recorded using both the visual analogue scale (VAS) and verbal rating scale (VRS). For the VAS rating, the patient set her/his sensitivity level by pointing with a pen to the colored 10-cm scale, and the distance from the border corresponding to an absence of pain was recorded. Tooth sensitivity was also scored according to the VRS, where 0=none, 1=mild, 2=moderate, 3=considerable, and 4=severe. Tooth sensitivity was evaluated during the bleaching procedure and immediately after bleaching agent removal. Twenty-four hours after the procedure, patients recorded the maximum level of tooth sensitivity perceived during the first 24 h and the level of sensitivity at that time. The VRS defined the presence (score different from 0) or absence of tooth sensitivity in all assessments. This binary outcome (main outcome) was used to define the risk of tooth sensitivity. The color of the canines was measured using a spectrophotometer one week after each bleaching session and 30 days after the final session. All evaluations were performed by two evaluators who were blinded to the allocation assignment. For each time of evaluation, color changes were measured by ∆L*, ∆a* and ∆b* calculations based on baseline data. Delta E was calculated using the following equation: ∆E=[(∆L)^2^+(∆a)^2^+(∆b)^2^]^1/2^.

At the last color evaluation, participants indicated their smile perception. Prior to the second tooth bleaching section, participants answered five questions regarding his/her perception regarding the bleaching protocol used and tooth bleaching results. For each question, the participants provided one of the following scores: 1 – completely agree, 2 – partially agree, 3 – no opinion, 4 – partially disagree, and 5 – completely disagree.

### Statistical analysis

Data from the color evaluation (∆L*, ∆a*, ∆b* and ∆E) were individually subjected to 2-way repeated measures ANOVA (“time of evaluation” was used as the repetition factor) followed by Tukey’s *post hoc* test. The absolute and relative risks of tooth sensitivity to each bleaching agent were calculated, followed by determination of the 95% confidence interval. For each evaluation time point, the absolute risks of tooth sensitivity were compared between the treatments using Fisher’s exact test. Regarding the level of tooth sensitivity, the VAS data did not exhibit a normal distribution. Thus, VAS and VRS data were analyzed by the Mann-Whitney test to compare the bleaching agents.

Scores for each bleaching agent, based on questions evaluating participants’ perceptions on the tooth bleaching procedures and outcomes, were analyzed by the Mann-Whitney test. Changes in participant perceptions about his/her smile were calculated by subtracting the final values by the value measured at the baseline, and comparisons between the bleaching agents were performed by a t-test. All statistical analyses were performed with a significance level of 95%.

## Results

**Figure 1:**
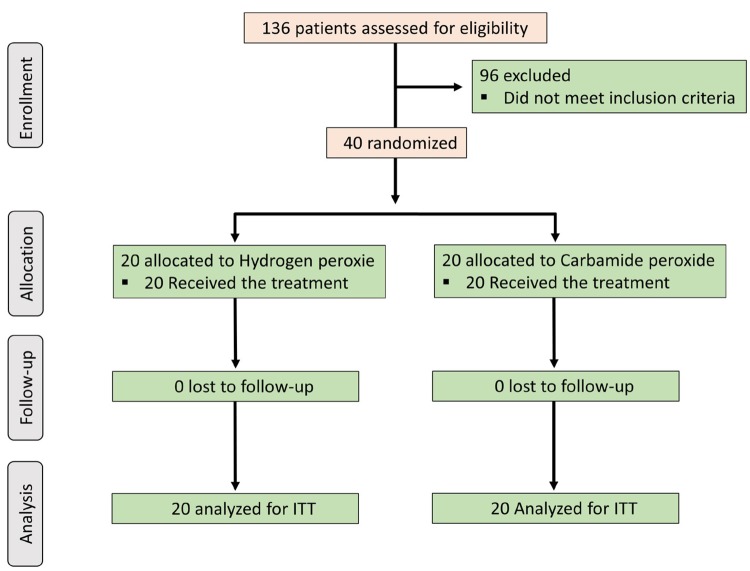
Flow diagram of the clinical trial

No difference (t-test, p=0.785) was observed in the age of patients allocated to receive tooth bleaching using hydrogen peroxide (23.5±4.5 years) or carbamide peroxide (23.8±3.5 years). Seventy percent of the participants allocated to receive tooth bleaching with hydrogen peroxide were females, whereas females represented 45% of the participants who had their teeth bleached with carbamide peroxide (Fisher’s exact test, p=0.514). Figure 1 presents the flow diagram of patients included in the study.

**Figure 2:**
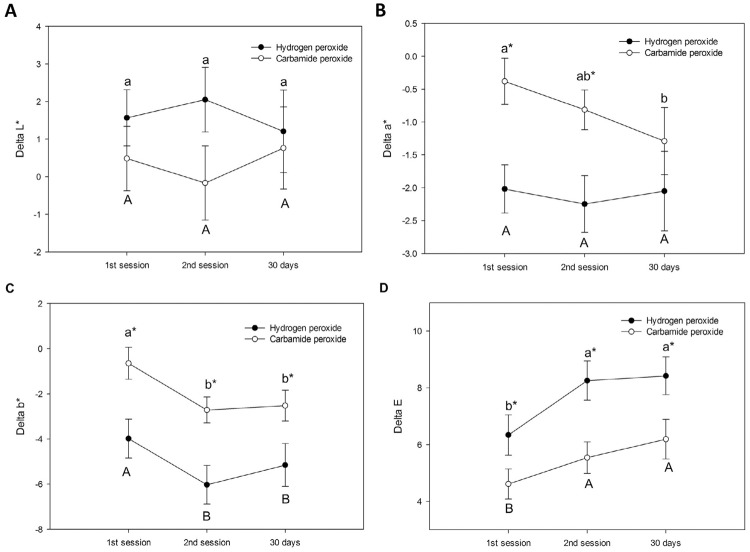
Results of the color evaluation regarding ∆L* (A), ∆a* (B), ∆b* (C) and ∆E (D). Distinct letters (lowercase for hydrogen peroxide; uppercase for carbamide peroxide) indicate significant differences between the assessment times for each bleaching agent (p<0.05). * Indicates a significant difference between the bleaching agents at each assessment time (p<0.05)

Results of the color evaluation are presented in Figure 2. “Bleaching agent” (p=0.273) and “time of assessment” (p=0.991) did not affect the ∆L*. The interaction between the factors was not significant (p=0.416). The “time of assessment” did not affect the ∆a* (p=0.159), but the effect of “bleaching agent” was significant (p=0.029; interaction, p=0.194). For all assessment times, hydrogen peroxide resulted in the highest ∆a* values. The factors “bleaching agent” (p=0.004) and “time of evaluation” (p<0.001) affected ∆b*; however, the interaction (p=0.800) was not significant. The highest ∆b* values were observed one week after the first session, with no differences between the other assessment times, regardless of the bleaching agent used. Hydrogen peroxide yielded greater b* value changes than carbamide peroxide at all evaluation time points. For ∆E, 2-way repeated measures ANOVA revealed that the “bleaching agent” (p=0.008) and “time of evaluation” (p<0.001) factors affected the results; however, the interaction between the factors was not significant (p=0.501). For both bleaching agents, the lowest ∆E values were observed after the first session, whereas no differences were noted at the other assessment times. Carbamide peroxide resulted in the lowest ∆E values at all evaluation times.

**Figure 3:**
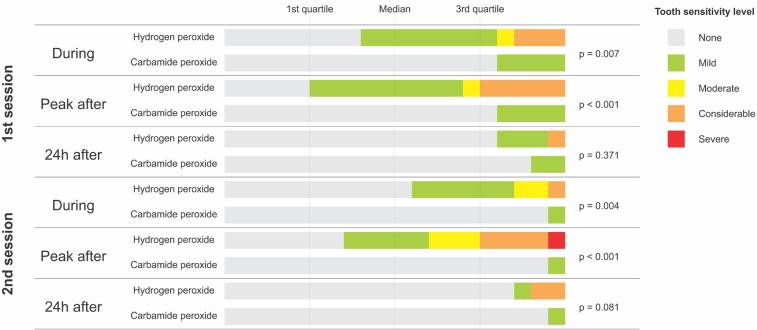
Distribution of the tooth sensitivity scores reported by patients using a verbal rating scale according to time of assessment and tooth bleaching protocol. * Calculated using the Mann-Whitney test

**Figure 4:**
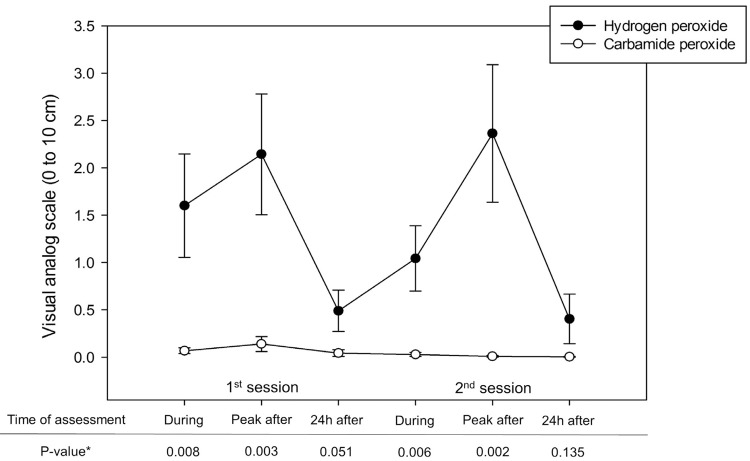
Mean (standard error) sensitivity levels reported by participants using the visual analog scale. * Calculated using the Mann-Whitney tes

Tooth sensitivity risk results are reported in Table 1. Irrespective of the tooth bleaching session, carbamide peroxide reduced the tooth sensitivity risk measured during the bleaching procedure compared with that following treatment with hydrogen peroxide. However, no difference in tooth sensitivity risk was noted between the bleaching agents 24 h after each bleaching session. Figure 3 presents the tooth sensitivity results measured using the VRS. Irrespective of the tooth bleaching session, carbamide peroxide resulted in a lower level of sensitivity, except when the measurement was performed after 24 h. The sensitivity results reported through the VAS are presented in Figure 4. Regardless of the bleaching session, tooth bleaching with hydrogen peroxide resulted in a higher level of tooth sensitivity during and following the procedure. However, no differences in sensitivity between the bleaching agents were noted 24 h after each session.

**Table 1:** Risk of patient-reported tooth sensitivity

Time assessment	Bleaching agent	Tooth sensitivity during treatment (number of participants)		p-value *	Absolute risk	Relative risk**
		Yes	No		(95% CI)	(95% CI)
During 1^st^ session	Hydrogen peroxide	12	8	0.022	0.60 (0.39-0.78)	0.33 (0.13-0.86)
	Carbamide peroxide	4	16		0.20 (0.08-0.42)	
24 h after 1^st^ session	Hydrogen peroxide	4	16	0.661	0.20 (0.08-0.42)	0.50 (0.10-2.43)
	Carbamide peroxide	2	18		0.10 (0.03-0.30)	
During 2^nd^ session	Hydrogen peroxide	9	11	0.008	0.45 (0.26-0.66)	0.11 (0.02-0.78)
	Carbamide peroxide	1	19		0.05 (0.01-0.24)	
24 h after 2^nd^ session	Hydrogen peroxide	3	17	0.605	0.15 (0.05-0.36)	0.33 (0.04-2.94)
	Carbamide peroxide	1	19		0.05 (0.01-0.24)	

* Fisher’s exact test; ** Hydrogen peroxide was used as a control for the relative risk calculation; CI - Confidence interval

Patients’ perceptions regarding the bleaching procedure and their own smiles are presented in Table 2. No differences were observed between bleaching agents regarding the comfort of procedures performed previously and during the tooth bleaching. A lower sensitivity than expected was observed primarily in participants who had their teeth bleached with carbamide peroxide. In contrast, a higher concordance was noted when teeth became whiter than expected, and increased satisfaction with the tooth bleaching results was observed when hydrogen peroxide was used. Participants allocated to receive hydrogen peroxide also reported more improvement in their smile after tooth bleaching.

**Table 2:** Responses to questions evaluating patients’ perception of the tooth bleaching procedures performed

Medians (1st / 3rd quartiles) obtained for the following questions (scores 1 to 5)			
Scores: 1 – completely agree, 2 – partially agree, 3 – no opinion, 4 – partially disagree, 5 – completely disagree.	Hydrogen peroxide	Carbamide peroxide	p-value*
Did you feel that the procedures performed prior to tooth bleaching were comfortable?	1 (1.00/1.00)	1 (1.00/1.00)	0.164
Did you feel comfortable during the tooth bleaching procedure?	1 (1.00/2.00)	1 (1.00/2.00)	0.759
Did you feel that the tooth sensitivity caused by bleaching was lower than expected?	1.5 (1.00/2.75)	1 (1.00/1.00)	0.022
Did you think the tooth bleaching resulted in whiter teeth than expected?	2 (1.00/2.00)	3.5 (2.00/4.00)	0.006
Are you satisfied with the results achieved with tooth bleaching?	1 (1.00/2.00)	2 (2.00/4.00)	0.004
**Means (standard deviation) of smile perception changes (VAS - 0 to 10 cm)**			
	Hydrogen peroxide	Carbamide peroxide	p-value**
How do you classify your smile? 0 – not attractive to 10 – completely attractive	3.09 (1.83)	1.28 (1.29)	< 0.001

VAS – visual analog scale. * Mann-Whitney Test; ** T-test

## Discussion

The tooth sensitivity reported by patients subjected to in-office bleaching remains the main concern related to this procedure^14^ ^,^ ^21^. Several approaches have been used by clinicians to reduce this adverse effect, including preemptive use of desensitizers^3^ ^,^ ^26^ or anti-inflammatory drugs^10^ ^,^ ^24^ ^[Bibr B25],^ ^26^. The use of low-concentration peroxides can also be a promising alternative for painless in-office tooth bleaching if the pain response is strongly related to an inflammatory process caused by the presence of peroxides and sub-products in the pulp tissue^4^ ^,^ ^14^ ^,^ ^18^. Carbamide peroxide-based bleaching agents are commonly used for at-home techniques using customized trays at concentrations ranging from 10 to 22%^8^. In the present study, a 37% carbamide peroxide bleaching agent was used in-office during a single 40-min application and resulted in reduced tooth sensitivity (both risk and level). However, the bleaching effectiveness was reduced compared with that of the procedure performed with 35% hydrogen peroxide. Thus, the hypothesis of the study was accepted.

In contrast to at-home bleaching procedures in which filled trays are used by patients for longer times (up 8 h)^5^ ^,^ ^8^, the 37% carbamide peroxide-based bleaching agent remained in contact with the buccal surface of the teeth for 40 minutes in this study. A previous study evaluating the kinetic release of hydrogen peroxide demonstrated that approximately 70% of its release occurred within 40 minutes when a 30% carbamide peroxide agent was analyzed^29^. Therefore, a significant bleaching effect could be expected within this time. Otherwise, bleaching performed with hydrogen peroxide yielded improved color changes compared with the results of treatment with carbamide peroxide. The differences in bleaching effects between the agents were mainly related to reductions of redness (b*) and yellowness (a*), whereas no differences in lightness (L*) were observed. In fact, a slight effect on lightness was observed for both bleaching agents used, whereas reductions in b* values were the main factor contributing to ∆E. It is reasonable to relate the reduced bleaching effect achieved with 37% carbamide peroxide to lower ultimate hydrogen peroxide concentrations, whereas the breakdown of this bleaching agent results in hydrogen peroxide levels of approximately 12%^29^. Interestingly, a recent clinical trial identified no difference in ∆E between 6% (approximately half the amount expected in 37% carbamide peroxide) and 35% hydrogen peroxide after two sessions of in-office tooth bleaching with two 12-min applications per session^18^. Thus, the differences between the bleaching agents in the present study can be further explained by differences in the presentation of agents evaluated.

The 35% hydrogen peroxide bleaching agent used is provided in two separate bottles. One bottle contains the peroxide, and the other contains an activating gel component. Furthermore, to increase the peroxide viscosity, the latter component also increases the pH of the mixed bleaching agent, thereby increasing the peroxide decomposition rate^5^ ^,^ ^12^. However, the 37% carbamide peroxide is available in a single syringe and does not require any mixing procedure before its application over the teeth surfaces. Therefore, similar to at-home procedures using filled trays, the reduction in peroxide pH caused by contact with the saliva is important to effectively break down the carbamide peroxide into hydrogen peroxide and urea and to increase the decomposition rate of the generated hydrogen peroxide^12^ ^,^ ^13^. However, the placement of cotton rolls before the bleaching procedure reduced the contact of saliva with the carbamide peroxide, hindering its breakdown and possibly reducing the concentration of free radicals available to oxidize the tooth structure. Therefore, the presentation of carbamide peroxide and reduction of contact between this agent and the saliva during the bleaching procedure can help to explain the reduced bleaching effect observed when this agent was used. The possible advantage of the direct application of a bleaching agent over the teeth without prior isolation with a gingival barrier could be associated with the reduced time required for the procedure. Moreover, in contrast to hydrogen peroxide, a single application of carbamide peroxide without the need for gel replacement results in further reductions in chair time. However, the patients did not report any difference between the protocols used in the present study regarding the comfort of procedures performed before and during the tooth bleaching. Furthermore, three patients reported gingival burn due to gum contact with high carbamide peroxide concentrations.

It is important to emphasize that the bleaching procedures performed with carbamide peroxide achieved greater than 5 units of ΔE, which is the threshold for the bleaching to be considered effective^2^. In fact, although the bleaching effect was lower than that expected for most patients who undergo tooth bleaching with carbamide peroxide, at least half of the patients (median score of 2) were satisfied with the results achieved with tooth bleaching. When the patients who received tooth bleaching with hydrogen peroxide were evaluated, more than 75% (score of 2 in the 3^rd^ quartile) agreed that the bleaching procedures yielded whiter teeth than expected or achieved satisfactory results. The best perceptions of treatment results were noted in patients who underwent tooth bleaching with hydrogen peroxide according to smile perception data. The average smile perception score following bleaching with hydrogen peroxide was 2-fold higher than that observed for carbamide peroxide.

Despite the reduction in bleaching effect, the presence of fewer reactive oxygen species from peroxide breakdown also reduces the negative effects of in-office bleaching related to tooth sensitivity^30^. During the bleaching procedures, the use of carbamide peroxide reduced the tooth sensitivity risk from 67 to 89% when compared with the risk associated with using hydrogen peroxide. Regarding the level of tooth sensitivity reported by patients, data on carbamide peroxide assessed by the VRS demonstrated that the 3^rd^ quartile experienced no sensitivity regardless of the assessment time. These data indicate that more than 75% of patients did not report any level of tooth sensitivity. Moreover, the VAS demonstrated levels of tooth sensitivity close to zero when carbamide peroxide was used. The sensitivity reported by patients following tooth bleaching procedures is related to inflammatory processes induced by the presence of peroxide and its products in the pulpal chamber, reducing pulpal cell proliferation, metabolism, and viability, and compromising the pulp-reparative capacity^6^ ^,^ ^17^ ^,^ ^30^. Thus, it is reasonable to associate lower concentrations of peroxide and its products with reduced tooth sensitivity levels and risk, in accordance with the findings of this study.

Regardless of the reported increased sensitivity and worse scores attributed to the question about tooth sensitivity to hydrogen peroxide, an important observation was that more than 75% (3^rd^ quartile was lower than 3) of the participants with teeth bleached by this agent did not disagree that the sensitivity experienced was lower than that expected. In fact, the average level of tooth sensitivity reported using the VAS was approximately 2 cm (maximum was 10 cm), and only one patient reported severe sensitivity (in the second session). Thus, despite the high risk of sensitivity observed for in-office bleaching with high concentrations of hydrogen peroxide (approximately 63% according to a previous systematic review), the level of this sensitivity is relatively low (mean of 2.8 cm on the VAS)^28^. Moreover, even when moderate to severe pain is present, tooth sensitivity tends to be significantly reduced or disappear 24 h after the bleaching procedure.

The findings of this clinical trial demonstrated that high concentrations of carbamide peroxide effectively achieve satisfactory bleaching effects when used in a single 40-min in-office application. In addition, despite a reduced color change compared with that resulting from the use of 35% hydrogen peroxide, 37% carbamide peroxide significantly reduced both the risk and level of the tooth sensitivity reported by patients. As sensitivity is the main concern of most patients who undergo tooth bleaching, products promoting pain reduction must be considered in any bleaching protocol. Regarding the reduced bleaching effect, the addition of any activating component to 37% carbamide peroxide could increase the pH solution and improve its bleaching effect. Therefore, further studies are necessary to confirm this last assumption.

## Conclusions

In-office tooth bleaching using 37% carbamide peroxide in a single 40-min application resulted in reduced risks and tooth sensitivity level with values close to zero, despite the reduced color change when compared with that achieved using 35% hydrogen peroxide.
